# SWOT Analysis of Communicable Disease Surveillance in Sudan

**DOI:** 10.1002/puh2.70024

**Published:** 2025-03-21

**Authors:** Alhadi Khogali, Rahaf AbuKoura, Nada Abdelmagid, Mona Ibrahim, Ruwan Ratnayake, Maysoon Dahab

**Affiliations:** ^1^ Said Business School and Nuffield Department of Primary Care Health Sciences University of Oxford Oxford UK; ^2^ London School of Hygiene and Tropical Medicine, Department of Infectious Disease Epidemiology and International Health Faculty of Epidemiology and Population Health London UK; ^3^ Sudan Research Group London UK; ^4^ Department of Social Policy and Intervention University of Oxford London UK

**Keywords:** communicable diseases, conflict, public health, Sudan, surveillance

## Abstract

Effective communicable disease surveillance is critical in Sudan to addressing the compounded health impacts of concurrent epidemics, health systems collapse and acute conflict. This article aims to map the strengths, weaknesses, opportunities and threats of Sudan's communicable disease surveillance systems before the current conflict to inform future health system rebuilding efforts.

Despite existing for 50 years, little is published on Sudan's disease surveillance systems. We conducted a scoping review to map the existing evidence on Sudan's surveillance systems and utilized a strength, weakness, opportunities and threats (SWOT) analysis to identify current and future gaps and opportunities to improve the performance of these systems for communicable diseases in Sudan. Our review shows that, prior to the conflict, disease‐specific surveillance and response activities were fragmented across various divisions of the Federal Ministry of Health, hindering a clear national‐level hierarchy. Sudan has committed to strengthening its disease surveillance system as part of its national health sector policy. Efforts to bolster pandemic preparedness and response were and continue to be recognized as critical. Chiefly among them is the need to invest in a fit‐for‐purpose national surveillance system that can operate against a background of acute crisis. Greater transparency and data sharing, clear guidelines for communication and collaboration and a centralized data management system can enhance the effectiveness of Sudan's communicable disease surveillance systems. Investment in a consolidated national surveillance system can support more efficient and coordinated responses to outbreaks and other health emergencies, with a view to future health system reconstruction.

## 1 Introduction

Sudan has faced a multitude of concurrent health emergencies resulting from its susceptibility to natural and man‐made disasters. These included refugee and displacement crises which have heightened the risk of frequent disease outbreaks [1]. Sudan also faced ethnic tensions, armed conflict and political instability, including the most recent armed conflict in April 2023, which led to a near health system collapse [2]. This underscores the urgent need for strengthening existing but disrupted disease surveillance systems as well as addressing gaps in population coverage, implementation and outbreak and emergency response. We conducted a strength, weakness, opportunities and threats (SWOT) analysis to highlight key gaps and opportunities of the current communicable disease surveillance system.

Disease surveillance is a critical component of public health as it provides the necessary information to understand disease distribution, trends and impact. This is essential in guiding public health interventions, identifying emerging threats and evaluating the effectiveness of interventions. Similar to many other surveillance systems worldwide, the surveillance of communicable diseases in Sudan employs a combination of two conventional approaches: indicator‐based surveillance (IBS) and event‐based surveillance (EBS) [3, 4]. IBS for communicable diseases has been in place in Sudan since the 1970s and was restructured in 2001 [3]. It has a primary function of promptly detecting and then reporting any suspected cases of an epidemic‐prone disease from a list of 26 diseases, categorized by priority and urgency for immediate and/or weekly reporting. IBS is predominantly sentinel‐based and operates as passive surveillance. However, during epidemics or outbreaks, it transitions into a partially active surveillance approach [5]. EBS in Sudan has been centrally coordinated through the Surveillance and Information Department (SID) of the Health Emergency and Epidemic Control (HEEC) General Directorate since 2016 as a complementary system for IBS. EBS components include several sub‐systems operating under the supportive activities unit, with a focal person for each and a coordinator for all EBS activities [5]. Although some scattered mortality records of specific diseases (e.g., HIV) or categories (maternal mortality records) are available, the information on any currently operating comprehensive mortality surveillance system remains limited.

Countries must prioritize the improvement of the quality, structure and performance of their disease surveillance systems. However, in the context of Sudan, the challenges of under‐reporting and lack of programmatic and empirical documentation present a significant challenge to health policy and decision‐making. Furthermore, academic records, national reports and evaluation documents on disease surveillance in Sudan are dispersed and difficult to access. Therefore, we aimed to conduct a scoping review to compile and map the existing evidence on Sudan's surveillance systems and identify current and future gaps and opportunities to improve the performance of these systems for communicable diseases in Sudan. The SWOT tool is used to classify barriers and facilitators of disease surveillance and to identify areas of improvement [6]. It has previously been employed to describe communicable disease surveillance in other countries and other parts of the healthcare system in Sudan. This is to identify key opportunities for improvements in the post‐conflict recovery period. These would not build only from weaknesses in the system but also key strengths. This is especially to maximize post‐conflict recovery efforts by capitalizing on the key strengths and opportunity areas. The methods are outlined in Annex I.

## 2 SWOT Analysis

### 2.1 Strengths

Sudan's disease surveillance systems have been functioning for nearly half a century [3], suggesting an established system with a comprehensive understanding among its stakeholders of their diverse functions and operations. This system has EBS systems and community‐based surveillance systems, point of Entry, Early Warning, Alert and Response Networks (EWARN) and communicable disease sentinel surveillance systems for numerous diseases (acute bacterial meningitis, haemorrhagic fevers, HIV, malaria, influenza, measles, acute flaccid paralysis and COVID‐19). These sub‐systems tend to feed into the Integrated Disease Surveillance and Response (IDSR) system, which links to the rapid and localized response.

Furthermore, the long‐standing disease surveillance systems in Sudan have contributed to improved outbreak response in the country (e.g., chikungunya outbreak response in 2020 [7]). The established and functioning surveillance has contributed to rapidly responding to the outbreak. This could be attributed to the developed institutional experience of the FMOH, the cyclic nature of outbreaks and their geographical focus on certain regions which made the local communities more sensitive to the outbreaks.

### 2.2 Weaknesses

One of the major weaknesses of the surveillance system in Sudan is the scarcity of publicly available surveillance data and reports on the performance of the current surveillance programmes, making it challenging to determine whether it is achieving the intended objectives. Fragmentation of surveillance presents another significant challenge [5]. EBS, for example, is coordinated by the HEEC General Directorate at the FMoH, whereas Vaccine‐Preventable Diseases (VPD) surveillance falls under the responsibility of the Expanded Programme on Immunization department at the Primary HealthCare (PHC) General Directorate [8]. This could be justified by the parallel governance and financing structures supporting each surveillance system (e.g., the disease‐specific funding from Gavi). This siloed approach contradicts the imperative for rapid and comprehensive epidemic surveillance for disease control, leaving minimal funding/resources or interest in other epidemic‐prone diseases.

Mortality surveillance in Sudan is yet another underexplored and under‐reported of public health in Sudan, with scanty literature available to describe its scope. Except for the COVID‐19 pandemic, there is little information on the number of deaths resulting from previous outbreaks.

### 2.3 Opportunities

Nonetheless, there are several opportunities for the current surveillance system in Sudan to improve given the advent of the COVID‐19 pandemic, which has resulted in an increased global commitment to enhancing surveillance systems and procedures [9]. Sudan also intends to meet these global commitments to strengthening its disaster information system and expanding its disease surveillance system [1]. This commitment will likely be well supported by donors, given their current interest in helping pandemic preparedness and response (PPR) efforts (e.g., World Bank Pandemic Fund). Sudan's government could certainly use this opportunity to invest in a robust national surveillance system tailored to support the country's specific needs. It is worth mentioning that it is unclear whether the spike in armed conflict would allow these commitments to flourish, given the catastrophic impact of donor withdrawal on health system development.

The Sudanese communities themselves have demonstrated a proactive role in detecting and managing outbreaks, organizations like ‘Nafeer’ exist in Sudan to support the community response to health emergencies and floods in Sudan. Other examples include the COVID‐19 response in different parts of Sudan and haemorrhagic fever outbreaks in Eastern Sudan. This grassroots momentum presents an opportunity for the government to integrate community‐led into a comprehensive national surveillance system that could be funded through a pandemic fund. It also could be utilized efficiently to tackle the shortage and high turnover rates of surveillance staff.

Additionally, digital health interventions could enhance surveillance in Sudan by optimizing IBS using the District Health Information System software and integrating diverse data sources, like weather forecasts, to quickly detect and predict outbreaks [10]. For instance, the Electronic Public Health Emergency Management (ePHEM) software developed with WHO EMRO goes beyond EBS signals to incorporate all surveillance systems inside and outside the Ministry of Health. Piloted in Sudan, ePHEM is now utilized in numerous countries.

### 2.4 Threats

Sudan's current political and economic landscape presents significant challenges to any effort to build or rebuild the health system and improve healthcare service delivery, including disease surveillance. Since 2019, there have been several changes in health leadership within the MoH, which hindered the ability to create policy change within the MoH. The concurrent financial collapse in Sudan has had a noticeable impact on disease surveillance and outbreak response, affecting various social determinants of health that are essential for disease prevention. The budget allocation for surveillance and disease control programmes shrunk significantly over the last decade. Another major challenge is the multiple outbreaks that hit Sudan; health partners in Sudan are under continuous pressure to respond to outbreaks, including haemorrhagic fevers and cholera/acute watery diarrhoea, with limited resources and manpower. As much as these outbreaks present an opportunity for improved performance and lessons learned, they also strain the system and create a vicious cycle, putting the MoH in a continuous state of ‘containing the blaze’. The exhaustive nature of such outbreaks, mainly regarding finance and manpower [11], makes the system vulnerable to future outbreaks.

## 3 Conclusion

The data deficiency presents a challenge to researchers and policymakers seeking to monitor and evaluate the effectiveness of Sudan's disease surveillance systems and identify areas for improvement. However, given the tense context in Sudan, there is a need for sound EBS systems that link communities and rapid response to contain outbreaks quickly; further, the comprehensive integration of surveillance with response systems is crucial.

Our study described the disease surveillance system before the conflict. Uniquely, we have presented key strengths and opportunities to contribute to ongoing efforts to restart the system at national and state levels. This information will remain critical for setting a benchmark for future evaluations of the surveillance systems in Sudan in the post‐conflict recovery phase.

## Author Contributions

**Alhadi Khogali**: conceptualization; investigation; writing–original draft; methodology; writing–review and editing; formal analysis; project administration. **Rahaf AbuKoura**: conceptualization; writing–original draft; funding acquisition; methodology; validation; writing–review and editing; formal analysis; resources. **Nada Abdelmagid**: conceptualization; validation; writing–review and editing; formal analysis. **Mona Ibrahim**: conceptualization; validation; writing—review and editing; formal analysis. **Ruwan Ratnayake**: conceptualization; validation; writing–review and editing; formal analysis. **Maysoon Dahab**: conceptualization; funding acquisition; methodology; validation; writing—review and editing; formal analysis; project administration; supervision; resources.

## Ethics Statement

The authors have nothing to report.

## Consent

The authors have nothing to report.

## Conflicts of Interest

The authors declare no conflicts of interest.

## Data Availability

The authors have nothing to report.

## Annex I

### Methods

We conducted the scoping review using a search of key databases, including Medline, Cochrane Library and EMBASE. We also searched Google Scholar for grey literature. We used the keywords described in Table 1, which yielded 2370 records. We also searched the Sudan Health Observatory (www.sho.gov.sd), a public clearinghouse for health information from the Ministry of Health, using Arabic and English search strings and purposively selected records describing disease surveillance systems in Sudan. The retrieved records were then compiled in the Rayyan platform (https://rayyan.ai/), where 519 duplicates were removed, and abstracts and executive summaries were assessed for inclusion, resulting in 43 full‐text articles selected for further screening. In each step, each record was independently assessed against the inclusion criteria by two reviewers in two stages to ensure the accuracy and consistency of the screening. Additionally, we reviewed the references of the included records to retrieve any additional records. The total number of records retrieved was 26.

**TABLE 1:** Key search terms.

(‘Surveillance’ OR ‘disease surveillance’ OR ‘outbreak surveillance’ OR ‘mortality surveillance’ OR ‘sentinel surveillance’ OR ‘mass gatherings surveillance’ OR ‘active surveillance’ OR ‘passive surveillance’ OR ‘indicator‐based surveillance’ OR ‘event‐based surveillance’ OR ‘community‐based surveillance’ OR ‘early warning’ OR ‘rumor surveillance’ OR ‘mortality surveillance’ OR ‘epidemic surveillance’ OR ‘communicable disease surveillance’ OR ‘alert management’ OR ‘public health response’)
AND
(‘Sudan’ OR ‘Khartoum’ OR ‘Darfur’ OR ‘Kordofan’ OR ‘Eastern Sudan’ OR ‘Kassala’ OR ‘Gadarif’ OR ‘Sinnar’ OR ‘Gazira’ OR ‘White Nile’ OR ‘Blue Nile’ OR ‘River Nile’ OR ‘Western Sudan’ OR ‘Southern Sudan’)
AND
(‘COVID‐19’ OR ‘malaria’ OR ‘cholera’ OR ‘chikungunya’ OR ‘rift valley’ OR ‘polio’ OR ‘dengue fever’ OR ‘influenza’ OR ‘yellow fever’ OR ‘communicable diseases’ OR ‘infectious diseases’)
